# Supervision, support and mentoring interventions for health practitioners in rural and remote contexts: an integrative review and thematic synthesis of the literature to identify mechanisms for successful outcomes

**DOI:** 10.1186/1478-4491-12-10

**Published:** 2014-02-13

**Authors:** Anna M Moran, Julia Coyle, Rod Pope, Dianne Boxall, Susan A Nancarrow, Jennifer Young

**Affiliations:** 1 Centre for Inland Health, Charles Sturt University, PO Box 789, Albury, NSW 2640, Australia; 2 School of Health and Human Sciences, Southern Cross University, PO Box 157, Lismore, NSW 2480, Australia

**Keywords:** Supervision, professional development, synthesis, mechanism, health practitioner, rural

## Abstract

**Objective:**

To identify mechanisms for the successful implementation of support strategies for health-care practitioners in rural and remote contexts.

**Design:**

This is an integrative review and thematic synthesis of the empirical literature that examines support interventions for health-care practitioners in rural and remote contexts.

**Results:**

This review includes 43 papers that evaluated support strategies for the rural and remote health workforce. Interventions were predominantly training and education programmes with limited evaluations of supervision and mentoring interventions. The mechanisms associated with successful outcomes included: access to appropriate and adequate training, skills and knowledge for the support intervention; accessible and adequate resources; active involvement of stakeholders in programme design, implementation and evaluation; a needs analysis prior to the intervention; external support, organisation, facilitation and/or coordination of the programme; marketing of the programme; organisational commitment; appropriate mode of delivery; leadership; and regular feedback and evaluation of the programme.

**Conclusion:**

Through a synthesis of the literature, this research has identified a number of mechanisms that are associated with successful support interventions for health-care practitioners in rural and remote contexts. This research utilised a methodology developed for studying complex interventions in response to the perceived limitations of traditional systematic reviews. This synthesis of the evidence will provide decision-makers at all levels with a collection of mechanisms that can assist the development and implementation of support strategies for staff in rural and remote contexts.

## Introduction

An important goal of health services is to provide accessible, equitable and efficient health care. The delivery and organisation of rural health care has seen significant changes over the past decade [1]. These changes have dictated the need for a rural health workforce that is adaptable and equipped with the skills and knowledge to diversify service delivery models [2].

However, the ability of rural health services to support adequately skilled and adaptable health practitioners is hampered by a number of factors including: poor recruitment and retention of practitioners [3]; shortages of practitioners [3]; high clinical loads, particularly for sole practitioners [4]; limited access to formal mentoring or supervisory relationships [4]; poor relationships with management [4,5]; difficulty accessing professional development activities or continuing education [4,5]; limited access to relief to allow professional or service development [4,6]; limited career pathways; limited or no dedicated work time allocated for professional reading or study [7]; and new graduates and sole practitioners possessing limited skills in service development [6].

The positive impact of the content and educational techniques utilised for continuing medical education (CME) interventions [8-10], training interventions [11], supervision [12] and mentoring [13] on the competence of the health workforce has been demonstrated elsewhere. It is less clear, however, how and to what extent the contexts in which such interventions are delivered contribute to their effect on staff, service and even patient outcomes.

This is particularly the case for supporting health practitioners in rural and remote contexts where accessing, delivering and participating in appropriate support opportunities is influenced by the complexity of the rural and remote environment [14-16]. Professional or geographic isolation, lack of financial resources and the costs of travel, time away from work, and cover and relief are common factors limiting support for practitioners in rural and remote areas. As Cameron *et al*. (p. 6) summarise: ‘Geographical location makes professional development a challenge, while isolation makes it essential for professional growth and peer support’ [16].

Recent evidence shows that a therapist’s decision to locate to, stay or leave a rural community is influenced by the availability of and access to practice supports, opportunities for professional growth, organisational commitment to supporting the practitioner and understanding the context of rural practice [17]. The need for more research to evaluate the effect of access to relevant continuing professional development (CPD) (as a form of support) on staff retention and, ultimately, rural health-care outcomes has been acknowledged [18] in particular by the World Health Organisation (WHO). The WHO’s policy of improving retention of rural health-care workers recommends that governments ‘design continuing education and professional development programmes that meet the needs of rural health workers and that are accessible from where they live and work, so as to support their retention’ [19].

However, the relation between the rural and remote context in which a support strategy is implemented and the mechanisms that facilitate or hinder the effect a support strategy can have on staff, services or patient outcomes has been poorly explored. The limitations of more traditional systematic review approaches in exploring issues in rural and remote health-care contexts have been established [20]. Therefore, using an integrative review and thematic synthesis of the literature, the aim of this paper is to identify: the range of support interventions reported in the literature for health-care practitioners in rural and remote contexts; how the success of support interventions is measured and defined; and the mechanisms that may contribute to the success of these interventions in rural and remote contexts. The use of an integrative review expands the variety of research designs that can be incorporated within a review’s inclusion criteria and allows the incorporation of both qualitative and quantitative information [21].

For the purpose of this paper, we have chosen to use the term support to encompass a number of concepts that can be seen as models of professional support. Namely, we were interested in exploring support in terms of supervision, mentoring, professional development and more general support interventions (for example, the provision of locum relief, support from colleagues and networks of practitioners [22]). These concepts are considered potentially modifiable factors that can contribute to a health-care practitioner’s decision to leave or stay in rural practice [23]. The term professional support has also been recently utilised to examine the utility of a professional support framework that encompasses a suite including professional supervision, mentoring, peer group supervision, peer review, work shadowing, in-service programmes and journal clubs (p. 562) [24].

We acknowledge that traditional definitions of several of these concepts overlap with one another. Both supervision and mentorship, for example, can be seen as models of professional support. Hence, the scope of the interventions explored in this review is deliberately wide; however, the population (or contextual) focus, that of rural and remote health practitioners, is relatively narrow.

We envisage that by exploring the broader concept of support, we will identify an appropriate suite of mechanisms to support health practitioners in rural and remote contexts.

## Methods

### Inclusion and exclusion criteria

Articles were included in this review if they empirically explored any intervention that was aimed at supporting health professionals in a rural or remote context. Specifically, the concept of support was explored in terms of support, supervision, professional development and mentoring (see Table 1 for a full list of search terms utilised). We limited our search to the period 1999 to 2012 as technological advances made since 1999, such as the development of the internet and laptops, have introduced new contexts in which support interventions for rural and remote practitioners are delivered, which we were keen to explore. Only those articles published in English language literature were included.

**Table 1:** Article identification process

**Process**	**Detail**
Sampling strategy	Selective: Sample databases from medicine, nursing, allied health and social science fields within specified limits
Type of study	All qualitative research (grounded theory, ethnography, action research, exploratory approaches, phenomenology), quantitative research (randomised controlled trials, controlled clinical trials, controlled before and after studies, uncontrolled before and after studies) and systematic reviews
Approaches	Subject searches, citation searches, contact with authors
Range of years	Beginning of 1999 to end of 2009. Updated in 2013 to include beginning of 2010 to end of 2013.
Language	English
Inclusion and exclusions^a^	Inclusion: Empirical research study of an intervention aimed at supporting^b^ health professionals; involves rural and remote populations; report evidence of outcomes related to staff, service or patients.
	Exclusion: No abstract for review, article is a commentary piece, or editorial.
Terms used	Mentor + health + rural OR remote
	Professional support + health + rural OR remote
	Supervision + health + rural OR remote
	Professional development + health + rural OR remote
	Continuing professional education + health + rural OR remote
	Continuing medical education + health + rural OR remote
	Preceptorship + health + rural OR remote
	Medical + supervision + rural OR remote
	Allied health + rural OR remote
Electronic sources	CINAHL Plus, EBSCOhost Health, Informit, MEDLINE OvidSP, Cochrane Library, SCOPUS, ISI Web of Knowledge, BioMed Central

^a^Detailed in the decision tree of Table 2; ^b^support refers to professional development, supervision, mentoring, continuing education, assistance, encouragement and resources that contribute to clinical practice, service delivery and staff satisfaction.

No report was excluded based on the data evaluation system described below; however, the strength of a paper was considered when reporting findings. When screening papers for inclusion we relied solely on the use of the terms ‘rural’ and ‘remote’ by the authors of the papers, although we acknowledge that the terms ‘rural’ and ‘remote’ can be explicitly defined using a more formal classification [25]. Where available we have detailed the authors’ definitions of ‘rural’ and ‘remote’.

### Search strategy

Broad eligibility criteria were used to assist with problem identification [21] and the capture of the full extent of literature in this field. A research assistant (JY) searched the electronic, peer-reviewed literature for the period 1999 to 2012 using guidance from Booth [26]. Multiple databases were utilised in the search and are outlined, along with key search terms, in Table 1.

### Data evaluation

**Figure 1:**
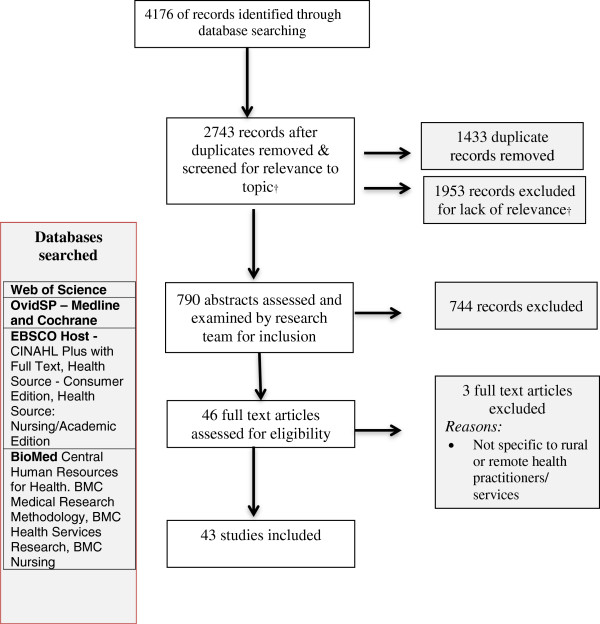
**PRISMA flow chart of the integrative review.** † abstracts screened using the following inclusion criteria: must have abstract for review; must contain reference to supervision, support (professional development/education), mentoring; must examine issues related to health care practitioners (and not undergraduate students); must be empirical research (not commentary, discussion or editorial); must be rural, regional or remote.

After removal of duplicates, an initial review of titles and abstracts produced 2,743 results (Figure 1). To better focus the review, 50 abstracts were randomly selected and jointly reviewed by two researchers (AM and JY). A joint decision was made as to which studies were relevant to the study aims and which were not, resulting in the construction of a preliminary decision process (outlined in notes accompanying Figure 1). This was used to screen the remaining references. By focusing the review, the number of potentially relevant sources was reduced from 2,743 to 790 papers.

Five researchers then independently screened an allocated set of abstracts using a pro forma for screening (Table 2), reflecting the inclusion criteria outlined in Table 1. This process resulted in a final set of 46 full text articles, which were read and assessed for eligibility for inclusion in the review (Figure 1). A total of 43 articles were included in the final review.

**Table 2:** Process for abstract screening

1. Does the paper relate to supervision, professional support or mentoring?	Yes – go to 2	No – exclude	Can’t tell – exclude
2. Does the paper describe a research study or evaluation (that is truly empirical)?	Yes – go to 4	No – go to 3	Can’t tell – go to 3
3. Is it a systematic review?	Yes – go to 4	No – consider for background	Can’t tell – exclude
4. Context			
Does the study describe: development of the intervention or model (input evaluation); implementation or actual intervention of the intervention or model (process evaluation); *or* evaluation of the intervention or model (impact evaluation)?	Yes – go to 5	No to all – consider for background	Can’t tell – consider for background
5. Outcomes			
Does the study analyse change in practitioner behaviour, service outcome or patient outcomes (within a qualitative, quantitative or mixed methods design?	Yes – go to 6	No to all – consider for background	Can’t tell – get full paper
6. Population			
Does the study examine rural and remote areas AND health practitioners?	Yes – **include**	No – exclude	Can’t tell – get full paper

The process for assessing quality is complex. Although a number of tools exist, there is no gold standard for calculating quality scores. The use of an integrative review with a thematic synthesis to extract a fuller understanding of ‘relationships, mechanisms and meaning’ within the evidence base [27] enables extraction of information from research that traditionally may not have been considered for review [21].

As we were primarily interested in qualitatively exploring and mapping the relations between the rural and remote context, the mechanisms of the support intervention and the outcomes of the support strategy, the magnitude of the effect of the intervention itself was not assessed. We therefore used thematic techniques to identify any evidence that linked mechanisms, specific to rural and remote contexts, to outcomes. As such, although the strength of evidence was examined, a quality assessment was not considered paramount to the identification of relations.

To broadly comment on the strength of the evidence utilised in this research, each study design was assessed according to its place in the research hierarchy using Daly *et al*.’s hierarchy of evidence for assessing qualitative research [28], the National Health and Medical Research Council (NHMRC) levels of evidence model for quantitative research [29] and mixed methods research was assessed using both Daly *et al*. and NHMRC levels of evidence for qualitative and quantitative components. Evidence hierarchies reflect ‘the potential of each study included in the systematic review to adequately answer a particular research question, based on the probability that its design has minimised the impact of bias on the results’ (p. 4) [29].

Given the mass of literature identified and the broad concepts explored, further additional hand searches of cited reference lists were not conducted nor were searches conducted within the grey literature or other sources.

### Data analysis

Thematic analysis techniques (see below) were then applied to the literature in conjunction with conceptual mapping using the mind-mapping software Freeplane to identify: the range of support interventions; the impact these interventions have on patient, staff and service outcomes in order to define ‘success’ and the mechanisms specific to rural and remote contexts within which the identified interventions were applied that may be associated with successful outcomes.

Freeplane allows the hierarchical, conceptual mapping of a range of related concepts reflecting synthesis approaches described by Baxter *et al*. [30] and Mays *et al*. [31]. Specifically, the thematic analysis approach employed is most closely related to framework analysis [32], which involves a systematic process of familiarisation with the data, identifying a thematic framework, indexing the themes, charting those themes into a hierarchical framework and then mapping and interpreting those themes.

## Results

This review identified 43 papers evaluating support strategies for the rural and remote health workforce (Tables 3 and 4). Papers were predominantly from Australia (*n* = 19), examining medical practitioners (*n* = 8) or nurses (*n* = 8) (Table 4).

**Table 3:** Summary of papers by intervention

**Citation**	**Design and quality**	**Participants and geographic location**	**Intervention and key contextual information**	**Outcome measures and findings**
**Papers examining supervision**				
Lynch and Happell [33]	Qualitative – ‘exploratory’ approach: document analysis and interviews	Nurses (in mental health)	Intervention: Examination of the ‘process and journey’ of a clinical supervision implementation strategy (part I) (face to face)	Primary measure: Service and staff outcomes – factors identified that led to successful implementation of clinical supervision models
		Rural: Service examined has 3,000 registered clients covering 44,000 km^2^	Contextual information: Five key stages of implementation were identified: Stage 1 – assessing the organisational culture and exploration of possibilities. Stage 2 – initial implementation strategy (need for leadership via leadership group, addressing issue with organisational culture, engagement of external organisation to provide a four-day supervision course for practitioners (where participants had to contribute to the overall strategic plan) and a one-day course for supervisors. Stages 3 to 5 in second article (below)	Positive impact: Large change of culture within the mental health programme. The estimated 80% of people initially negative and suspicious about clinical supervision was now estimated to be only 15% to 30%. Considerations: Strategies for sustainability developed included: continuity of review programme and leadership team (working group) to oversee actions and to work with senior management.
	Level III	Australia		
Lynch and Happell [34]	Qualitative – ‘exploratory’ approach: document analysis and interviews	Nurses in (mental health)	Intervention: Examination of the ‘process and journey’ of a clinical supervision implementation strategy (Part II) (face to face)	As above
	Level III	Rural: Service examined has 3,000 registered clients covering 44,000 km^2^	Contextual information: Active involvement of staff in programme design and evaluation (dedicated ‘team’ of staff to undertake needs analysis (talking to staff, assessing workplace culture) and take control of decisions and implementation. External training for supervisors and supervisees in supervision; established a strategic plan; marketed the programme (official ‘launch’ of the programme, using a strategic plan to demonstrate organisational commitment); continual reflection and gathering of feedback; formal internal review of programme (demonstrated change, effect, impact on staff; clear leadership)	
		Australia		
English *et al*. [35]	Mixed methods – ‘following a thread’	Multi-disciplinary	Intervention: Secondary analysis of data examining how the ‘inputs’ of supervision, feedback and facilitation affected implementation of best practice (face to face)	Primary measure: Staff outcomes (qualitative) – skills, satisfaction, (change in) attitude, leadership
	Level I	Rural	Contextual information: External support and organisational commitment **(**external supportive supervision and local management and clear lines of communication regarding expectations established prior to programme); attributes of educator (facilitators were used within intervention hospitals); active involvement of stakeholders (‘health workers must not only know how to perform a task (for example, prescribing) but be willing to perform it’); networking and relationships (team working and integrated working associated with greater satisfaction)	Secondary measure: (qualitative) service outcomes – resource allocation, improved clinical systems
		Kenya		Positive impact: A multi- faceted intervention strategy can change provider behaviours and improve the quality of inpatient care across a range of high mortality, target diseases.
				Considerations: In all settings, health worker motivation was a challenge
Xavier *et al*. [36]	Non-experimental – descriptive pre-and post-intervention evaluation	Psychologists and social workers	Intervention: Training, education and supervision. Clinical supervision and education provided by videoconference from a tertiary metro teaching hospital with individual telephone supervision each month (non-face-to-face: real-time videoconferencing)	Primary measures: Staff outcomes – number of participants, self-reported knowledge gains, self-reported confidence in management of particular conditions; satisfaction with the programme
	Level IV	Australia	Contextual information: Externally organised and supported: site coordinators were available to offer technical assistance at the majority of the locations. An administrative assistant was employed to coordinate the study; needs analysis was undertaken prior to the event	Positive impact: Significant increases in self-reported confidence in the areas covered by the educational component, for example assessing and treating pain in people with cancer (Po0.01). Self-assessment of overall effectiveness in current management of psychological distress from pre- to post-evaluation increased by 25%. Participants indicated that attending the educational sessions increased their knowledge (mean 1⁄4 7.3 out of 10). With regard to telephone supervision, most (80%) were very or extremely satisfied. The feedback indicated that remote supervision was well received and that participants were keen to continue their involvement. Overall: It is feasible and acceptable to provide clinical supervision and education via videoconference
**Papers examining professional support**				
Conger and Plager [37]	Qualitative – phenomenology	Nurses	Intervention: Mechanisms promoting connectedness for masters students in rural areas were identified and explored (combination: face to face, telephone, email)	Primary measure: Staff outcomes – mechanisms that encouraged connectedness in rural areas
	Level II	Rural	Contextual information: Targeted development of support networks (relationships formed during study, other professionals in health centre, collaborative practice, mentoring); targeted development of relationship with large urban or metro health centres; targeted development of relationship with community; access to technology; avoiding mechanisms that promote disconnectedness (lack of relationships with health centres, poor avenues of communication with other health centres, lack of mentoring)	Positive impact: Connectedness enhanced by: development of support networks, relationships with large urban medical centres, availability of electronic communication and connections with the rural community. Graduates who reported a sense of disconnectedness when working in a rural community were less likely to remain in that community
		USA		Negative impact: mechanisms that promote disconnectedness such as: lack of relationships with other health centres or poor communication avenues with other health-care centres; lack of mentoring (incidentally felt phone calls not enough)
Teasley *et al*. [38]	Non-experimental – descriptive pre- and post-intervention evaluation	Nurses	Intervention: Nurses were requested to participate in meetings that generated and prioritised a list of interventions for implementation to improve perceptions of workload (face to face)	Primary measure: Staff outcomes – workload perceptions.
				Secondary measure: Staff outcomes – satisfaction and retention.
	Level IV	Rural Kentucky: Community of 5,000, 60 miles from major metropolitan areas	Contextual information: Active involvement of stakeholders in programme design and evaluation; active involvement of staff in change process	Positive impact: Participant engagement in developing and implementing self-identified work environment issues led to improved workload, work satisfaction and intent to remain.
		USA		
Cameron *et al*. [39]	Qualitative – collective case study methodology	Medical practitioners, community members, spouses	Intervention: Exploration of community factors that promote physicians to practice and remain in a rural area (face to face)	Primary measure: Staff outcomes – factors that support retention of practitioners (this is also identified as a community outcome)
	Level I	Rural	Contextual information: Active involvement of stakeholders (as evidenced by ‘active support’ theme); networking and relationships (connection and reciprocity themes)	Positive impact: Four themes emerged. Appreciation, connection, active support (for the practitioner and pursuits of the practitioner for example defending health region) and physical and recreational assets were positively related to physician retention. These community factors existed to different degrees but were present in all communities. Reciprocity was a fifth factor that emerged.
		Alberta, Canada		
Healey-Ogden *et al*. [40]	Qualitative – interviews	Nursing	Intervention: Implementation of an 80/20 staffing model whereby staff have 20% of salaried time off from direct patient care to pursue professional development activities (face to face)	Primary measures: Staff outcomes – retention, knowledge, personal growth Secondary measures: Service outcomes – team engagement, quality of care, collaboration
	Level III	Rural	Contextual information: Organisational commitment (senior management and other partners on steering committee); external support (university, funded by Ministry for Health); accessible and adequate resources with 20% of time for CPD, training or supervision made available through creation of backfill positions (nurses were paid for their 20% time off clinical duties and could access funding to pay for travel and courses and so on); leadership (project coordinator was hired and utilised); flexibility (timing often mismatched between availability of backfill and course availability)	Positive outcomes: 4,000 hours of professional development and learning activities; positive effect on personal growth and work environment; improved job satisfaction and (unmeasured) intention to remain in job; perceived increase in quality of care; increased collaboration with staff of other hospitals and universities; team development
		British Colombia, Canada		Considerations: Participants had scheduled professional development time during the summer, but most formal educational opportunities begin in September, hence professional development time and the availability of backfill staff did not always match; opportunities sparse in local area implies need for funding for travel and accommodation
**Papers examining training or education**				
Arora *et al*. [41]	Non-experimental – descriptive pre- and post-evaluation	Medical practitioners	Intervention: Use of a ‘telehealth clinic’ bringing together metro specialists and rural community based primary care providers to provide care to hepatitis C sufferers (non-face-to-face: real-time videoconferencing)	Primary measures: Service outcomes (from patient level) – efficiency, access and quality/completeness
				Secondary measures: Quality and completeness of health information and services received by clients
	Level IV	Rural	Contextual information: Needs analysis; external support; financial support (three-year funding grant); regular feedback and evaluation opportunities; accessible and adequate resources (two-day orientation to technology and format of sessions); networking and relationships (development of ‘knowledge networks’ between practitioners of different specialities); application of formal learning strategy (learning loops)	Positive impact: Uniform agreement by participants – benefit to the practice and patients, expanded access to specialists, and the provider’s professional enhancement; significant increase in competency sustained for >12 months; competent to educate others; perceived improvements in patient safety and quality of care
		New Mexico, USA		
Bennett-Levy *et al*. [42]	Experimental – randomised controlled trial	Multi-disciplinary (psychologists, social workers, nurses, counsellors, medical practitioners)	Intervention: Online training programme for rural and remote mental health practitioners in cognitive behavioural therapy (CBT) (non-face-to-face: internet, video clips)	Primary measures: Staff outcomes – CBT knowledge, skills, confidence, utilisation and satisfaction with programme
	Level II	Urban, regional, rural	Contextual information: External support; accessible and adequate resources (discounted access to online learning programme provided); networking and relationships (15-min support sessions provided by experienced psychologist after each online learning module completed)	Positive impact: Participants in both groups improved their performance scores from pre-program to post-program and follow-up; supported training group was more likely to finish or very nearly finish (96%) than the independent group (76%) (c2 = 3.93, *df* = 1, *P* < .05); program characteristics, including the program design and content, proved highly acceptable; value of the 15-min support sessions was almost unanimously endorsed by the supported training group
		Australia		
Blattner *et al*. [43]	Qualitative – thematic analysis of interviews	Nurses and medical practitioners	Intervention: Staff at a rural hospital were trained in using a newly installed point of care test analyser (face to face)	Primary measures: Staff outcomes – change in practice behaviour, job satisfaction, process facilitators and barriers Secondary measures: Service outcomes – sustainability of intervention
	Level III	Remote	Contextual information: Access to training, skills, knowledge for the intervention (including refresher courses in interpreting tests); accessible and adequate resources (point of care test analyser located on ward)	Positive impact: Training and use of point of care testing increased diagnostic certainty and improved confidence in clinical decision-making; transfer decisions could be made earlier than they otherwise would have been and often treatment could begin immediately; reduced need for inter-hospital transfers and increased discharge rate; higher standards of practice; access to continuing professional education (CPE)
		New Zealand		Negative outcomes: Workload increase – managing patients who would previously have been transferred and who now require more care; can be time-consuming; over-testing may become a problem
Brambila *et al*. [44]	Quasi-experimental – pre- and post-intervention and control groups	Health practitioners (*n* = 40)	Intervention: Train the trainer: snowballing of a training intervention where two practitioners from each health district (*n* = 20 × 2 trainers) undertook training in tools to improve service quality. They then each trained approximately six trainees per health district in the programme (face to face)	Primary measures: Service outcomes (from patient level) – efficiency, access, quality and completeness
				Secondary measures: Service and patient outcomes – quality and completeness of health information and services received by clients
	Level III-3	Rural: Approximate population served 580,000 individuals	Contextual information: External support, coordination and programme; structure and content of programme; train trainers how to use job tools to improve service quality; train trainers how to train health-care practitioners; motivational and attitudinal change elements built into curriculum; needs assessment (content of programme in response to problem areas); appropriate skills and knowledge	Positive impact: Access to services increased significantly
		Guatemala		No impact: No reduction in client waiting times or total time spent by clients at facilities
Buckley *et al*. [45]	Non-experimental – descriptive post-intervention evaluation	Nurses	Intervention: Digital photographs were used to develop treatment plans and assess competency of non-specialist nurses in wound management utilising specialist support (non-face-to-face: telephone, email and digital photography)	Primary measures: Service outcomes – agreement on wound assessment and wound management plan between specialist and non-specialist nurse
	Level IV	Rural	Contextual information: Access to technology (computer, internet, email, digital cameras, IT programmes); correct use of technology, ability to use technology (issues identified around ability to take the ‘right’ picture); information privacy (permission to transmit patient information via email); appropriate use and combination of technology to achieve desired outcomes (intervention needed both verbal and pictorial reporting to improve accuracy of reporting)	Positive impact: Agreement on more basic assessment parameters.
		USA		Less impact: On average there was poor agreement on more complex parameters. Verbal reports often missed vital signs leading to poor agreement between the specialist and non-specialists.
Church *et al*. [46]	Mixed methods – pre-, during and post-intervention questionnaire and focus groups	Multi-disciplinary	Intervention: Interprofessional education programme in mental health for practitioners in six rural communities (combination: face to face, videoconferencing)	Primary measures: Staff outcomes – satisfaction, knowledge, skills, confidence
				Considerations: Vision is necessary for accurate diagnosis, potentially not just of the wound but of the home environment also
	Level IV	Rural	Contextual information: External support (programme run and supported by researchers); networking and relationships (professionals from different systems brought together, structure of the programme – small groups, interactive, case-based learning)	Positive impact: Significant increase in confidence for seven of the eight mental health interventions and four of the six mental health issues that had been taught in the programme; more reflective mental health practice, more aware of mental health issues; integrating new knowledge and skills into their work; interprofessional referrals, interagency linkages and collaboration increased
		Rural Newfoundland and Labrador, Canada		
Cunningham *et al*. [47]	Qualitative – focus groups	Administrative and clerical staff	Intervention: Mechanisms contributing to effective protected learning time were identified (face to face)	Primary measures: Staff outcomes – satisfaction with, benefits of, advantages and disadvantages of PLT
	Level III	Rural	Contextual information: Organisational commitment; structured learning outcomes; structure of the programme (spending time with other teams and services, spending time with immediate colleagues, centrally organised events)	Positive impact: Useful to do with other teams and team members especially team-building activities Considerations: Increased workload the day after. Needs to include quality educational experiences. May be improved using a learning needs assessment
		Scotland, UK		
Doorenbos *et al*. [14]	Non-experimental – descriptive post-evaluation	Multi-disciplinary	Intervention: A series of cancer education sessions were delivered using telehealth technology to rural health-care providers (non-face-to-face: real-time videoconferencing)	Primary measures: Staff outcomes – satisfaction (content and mode), attendance rates
	Level IV	Rural	Contextual information: Active involvement of stakeholders (participants worked with university and clinical experts to develop cancer programme; participating rural health-care providers were also engaged in selecting topics and identifying convenient and feasible dates and times for the videoconference presentations); needs analysis; marketing the programme (the series was publicised and scheduled well in advance to allow providers to plan attendance at the presentations most relevant to them); accessible and adequate resources; external support (university technical staff hosted each presentation and were continually available for troubleshooting technological problems)	Positive impact: Overall satisfaction with telehealth as a mode of delivery; educational session information rated highly; high attendance rates; accessing CPE became a reality for rural health-care providers
		Washington State, rural Alaska, USA		
D'Souza [48]	Non experimental – cross-sectional questionnaire design post-intervention	Mental health practitioners and medical practitioners (general practitioners (GPs))	Intervention: Delivery of educational and clinical modules for mental health via telemedicine and videoconferencing facilities (non-face-to-face: real-time videoconferencing)	Primary measures: Staff outcomes – satisfaction with the service and associated outcomes; feelings of isolation, fulfilling of academic needs, relevance to professional development, effect on self-assessed competence with mental health clients
	Level IV	Rural	Contextual information: Access to technology; timing of delivery (during team meeting time); mode of delivery (videoconferencing); structure and content of programme (lecture notes delivered prior to videoconferencing, 60-minute CPD blocks plus interactive discussion time)	Positive impact: High satisfaction scores with the service fulfilling their professional and academic needs. The service helped improve confidence and competence in managing mental illness
		Australia		
Ellis and Philip [49]	Mixed methods – pre- and post-questionnaire and interviews	Multi-disciplinary	Intervention: Development, delivery and evaluation of a short course in managing mental health emergencies at rural and remote health sites (face to face)	Primary measures: Staff outcomes – skills, satisfaction, attitude
	Level IV	Rural and remote towns in South Australia, Northern Territory, Queensland and Western Australia	Contextual information: External support (conducted by Australian rural nurses and midwives using grant from Department of Health and Ageing); adequate and accessible resources (workbook provided to participants; course delivered in rural and remote sites; mode of delivery – face to face)	Positive impact: Significant improvement between pre and post mental health assessment skills (unmatched comparison); changed attitudes towards mental health; improved communication ability when dealing with mental health clients
Glazebrook *et al*. [50]	Non-experimental – pre- and post-test evaluation	Medical practitioners	Intervention: Outreach ultrasound education workshops held in rural locations – specialist doctors from metro areas delivered workshops with local sonographers (face to face)	Primary measures: Staff outcomes – pre- and post-workshop knowledge tests (unvalidated)
				Secondary: Self-rated levels of knowledge, confidence and expertise in ultrasound
	Level IV	Small rural hospitals	Contextual information: External support and organisation; local support (local experts utilised); funding and travel for outreach experts; structure and content of programme (face-to-face: hands-on workshops)	Positive impact: Significant improvement in knowledge and self-reported confidence with ultrasound by medical practitioners
		Australia		
Gorsche and Woloschuk [51]	Quasi-experimental – longitudinal, matched, case–control study	Medical practitioners	Intervention: Training programmes run within an ‘enrichment programme’ for rural and remote medics (mode not specified)	Primary measures: staff outcomes – goal attainment and retention.
	Level III-2	Rural: Any Alberta community more than 50 km from a major metropolitan centre	Contextual information: External support (initiative of the Alberta government); accessible and adequate resources (fully supported to undertake training of choice – for example preceptors were compensated and locums arranged)	Positive impact: 97% of participants achieved training or learning goals; all participants were using their new or upgraded skills at 5 years; after 5 years, 100% in the matched enrichment group remained in rural practice compared with 71% physicians who did not partake in the EP (RR = 1.31; confidence interval: 1.06 to 1.62; *P* < 0.05).
		Canada		Only paper to demonstrate a statistical link between supportive context, skill acquisition and retention of rural practitioners.
Haythornthwaite [52]	Non-experimental – descriptive pre- and post-intervention evaluation	Mental health practitioners	Intervention: Simultaneous videoconference sessions presented over 12 weeks (‘Rural Links’ programme). Included fortnightly training sessions accompanied by reading material on topics covered and workbooks for use in-session (non-face-to-face: multi-site real-time videoconferencing)	Primary measures: Staff outcomes – number of participants, knowledge in relation to the training topics, participants’ views of video conferencing as a training modality, participant satisfaction
	Level IV	Rural and remote	Contextual information: Access to technology; resources: workbooks and session exercises; assume externally organised	Positive impact: Varied significant improvements in knowledge gains for particular teaching modules (although not consistent gains for all modules); compared with metropolitan participants, who received face-to-face training, rural participants showed similar levels of improvement in learning for most areas; high levels of participant satisfaction with videoconferencing delivery and programme content
		Western Australia		
King *et al*. [53]	Qualitative – critical ethnographic post-intervention	Aboriginal health workers	Intervention: A post-graduate university course undertaken by Aboriginal health workers (developed for nurses and allied health practitioners) to qualify them as diabetes educators	Primary measures: Staff outcomes – perceptions of the course, development as a health practitioner, relevance of the course to self and clients, learning outcomes
	Level II	Rural and remote	Contextual information: Reflection, feedback, evaluating outcome of the course; the course has to be relevant and academically targeted appropriate to the participant	Positive impact: Undertaking a post-graduate diabetes education course can improve confidence and competence in Aboriginal health workers. Course helped the Aboriginal health workers become more confident and competent as health professionals and empowered to learn and impart new knowledge as a practitioner
		South Australia		
Kelley *et al*. [54]	Non-experimental – cross-sectional survey design	Palliative carers	Intervention: Information regarding how a training programme was developed, planned and delivered in collaboration with local community partners (face to face)	Primary measures: Staff outcomes – self-reported knowledge and skills of practitioners. Service outcomes – sustainability of the programme, development of palliative care programmes in other agencies or the community
	Level IV	Rural: ‘Towns and municipalities less than 10,000 population and located outside the commuting zone of urban centres larger than 10,000 population.’ Remote: ‘isolated community with limited resources, 80 km distance or four or more hours travel from a major urban centre of >50,000 population’	Contextual information: Course content was locally relevant; networking opportunities; train-the-trainer approach, emphasis within course material on training participants to translate their knowledge gains to co-workers	Positive impact: Increase in self-reported knowledge, confidence in practice and skills. Sustainable via participants imparting learnt knowledge via mentoring and workshops to co-workers. May increase number of palliative care programmes.
		Canada		Considerations: Networking and learning about supporting resources were identified as the most important elements of the programme; able to collaborate more as a group to improve services and act as a common voice. Indicated they shared information with other staff via mentoring, meetings, case conferences, formal in-service sessions, formal workshops.
				Ideal learning: Off site in nearby town, small groups, interactive
Koczwara *et al*. [55]	Non-experimental – descriptive planning phase and post-evaluation	Multi-disciplinary	Intervention: Development, implementation and evaluation of an online educational programme (oncology) for rural health practitioners	Primary measures: Staff outcomes – change in practice, satisfaction with programme, users (attendance)
	Level IV	Rural and remote	Contextual information: Needs analysis (survey and focus groups conducted with rural practitioners); regular feedback and evaluation opportunities; marketing of the programme (programme launch at national conference, online advertising to target audiences); accessible and adequate resources; networking and relationships; employ specific learning approach; skills to deliver the intervention (facilitator employed and trained in online environment and subject matter)	Positive impact: High attendance and completion rates; perceived change in practice as a result of completion of learning programme; learning needs met and achievement of specific learning goals; high satisfaction with online multimedia
		Australia		
Newman *et al*. [56]	Non-experimental – cross-sectional survey post-intervention	Multi-disciplinary	Intervention: Use of videoconference facility in different urban and rural settings to deliver a one-off education conference	Primary measures: Staff outcomes – knowledge, socialisation, information exchange, ease of use
				Secondary measures: Numbers of participants, geographic location
	Level IV	Urban, rural and remote	Contextual information: External support and organisation (technical preparation of videoconferencing was by the conferencing and media staff from the lead hospital or health service in liaison with staff and departments from other services; ‘site facilitators’ utilised at each site); adequate preparation (speakers provided with guidelines on etiquette and teaching methods); teaching rehearsals prior to event	Positive impact: Useful for learning and were able to contribute or be part of a learning community
		Australia		Less impact: Mostly a passive experience. Not overly easy to use
Schoo *et al*. [57]	Mixed methods – action research (questionnaire and interviews)	Physiotherapists	Intervention: Continuing education programme developed, implemented and evaluated by local physiotherapy practitioners with researchers from a university (face to face)	Primary measures: Staff outcomes – relevance, attendance of programme
				Secondary measures: Staff outcomes – perceived effect on clinical practice
	Level III (Daly)	Regional and rural: ‘Accessible and moderately accessible’ on the Accessibility/Remoteness Index	Contextual information: Location of programme (locally delivered); teacher attributes (highly qualified); needs analysis prior to programme development; active involvement of stakeholders in programme development and evaluation (identification of targets and measures for success prior to intervention, active participant engagement with institutional facilitation); external organisation, input and facilitation (needs assessment, development of programme, evaluation tools)	Positive impact: All targets were reached. Attendance – more than half (57.2%) of physiotherapists in the region attended a minimum of four sessions and 68.6% attended at least one ‘on-site’ workshop. More than two-thirds of the physiotherapists (68.6%) knew of others who attended at least one of the continuing education (CE) functions of the 2004/5 programme and 45.7% of these physiotherapists received useful information from others who attended. Interactive CE programme had a positive influence on perceived clinical skills
		Australia (ARIA)		
White *et al*. [58]	Non-experimental – cross-sectional survey post-intervention	Medical practitioners (GPs)	Intervention: Government-run CME workshops (face to face)	Primary measures: Staff outcomes – professional isolation, confidence, commitment to remain in rural practise (retention)
	Level IV	Rural: Rural Remote and Metropolitan Areas (RRMA) classification four to seven locations	Contextual information: Needs analysis; clinician-led content; funding (government department funded travel and accommodation); time relief (locum support or locum rebates available for more remote GPs)	Positive impact: Access to CME contributes to confidence in practicing in remote and rural areas; CME strongly alleviates professional isolation; less likely to remain in practice without access to CME
		Australia		
Wright *et al*. [59]	Non-experimental – descriptive pre- and post-evaluation	Medical practitioners	Intervention: Evaluation of an educational support programme for international practitioners practicing in rural areas (combination: simulated face-to-face consultations, workshops, weekly meetings, interactive web-based learning modules)	Primary measures: Staff outcomes – clinical practice and competency, retention (at three months post-intervention); satisfaction with the programme
	Level IV	Regional and rural: RRMA two to five	Contextual information: Needs analysis (via a pre-programme learning needs analysis); regular feedback and evaluation opportunities (post workshop and session evaluations and post programme evaluation); accessible and adequate resources	Positive impact: Needs assessment enabled participants to articulate specific skills and knowledge that would assist them to work more effectively in their current clinical contexts; statistically significant and positive changes were identified post-intervention for (i) technical skills appropriate to current practice; (ii) willingness and effectiveness when teaching or training colleagues and (iii) communication with carers and family. Satisfaction with the programme and development of a learning community in Gippsland
		Australia		
**Papers examining mentoring**				
Butcher [60]	Non-experimental – descriptive pre-post-evaluation	Nurses and dietitians	Intervention: Mentoring to upskill or train to become certified diabetes educators or simply to improve knowledge of diabetes (combination: face to face, telephone, email)	Primary measures: Service outcomes – access to quality diabetes services; staff outcomes satisfaction with programme
	Level IV	Remote: Population of 902,195 spread across 147,042 square miles: population density of 6.2 persons per square mile	Contextual information: Needs assessment (learning needs of all enrolled in programme were assessed and matched to course materials and a mentor); external support and coordination (central coordinator designated to programme); resources (lending library for study, mentoring manual for mentors and mentees); structure and content of the programme: mentoring was face to face, telephone and email; observation of mentor in diabetes management also encouraging; combination of mentoring programme structure, content and delivery modes (email, face to face, resources)	Positive impact: 30% of enrolled nurses and dietitians gained certification. Number of educators increased 47% (but unsure if directly related to intervention)
		USA		
Gibb *et al*. [61]	Qualitative – focus groups held before and after an action research intervention	Nurses	Intervention: Research officer worked with staff to develop a definition of mentoring, the results of which were converted into questionnaires by the research team eventually becoming a set of guidelines of desired qualities for mentors and mentees and an evaluation tool for monitoring the mentoring relationship (mode: n/a)	Primary measures: Staff outcomes – understanding of mentoring, key qualities in mentors and mentees, success of mentoring strategy
	Level III	Small rural hospitals	Contextual information: Needs analysis (staff perception of mentoring needs); external support (facilitation of action research by university); active involvement of stakeholders in programme design and evaluation (the act of coming to an understanding and a working definition of mentoring in context; action research enabled greater understanding of role of mentoring, which in turn allowed for effective mentoring relationships to develop); conversion of discussion into a questionnaire for evaluation and into a guideline document for mentoring	Positive impact: More structured mentoring practice
		Australia		Considerations: Qualities of a good mentor were identified, action research enabled greater understanding of role of mentoring which in turn allowed for effective mentoring relationships to develop. Link identified between mentoring and development of clinical competence. Key to successful mentoring was management support
**Papers examining a combination of support interventions**				
Dalton *et al*. [62]	Mixed methods – pre- and post-intervention evaluation	Pharmacists	Intervention: Education, training and mentoring. Online preceptor education programme with interactive learning modules and online interactive mentoring via discussion groups (non-face-to-face: real-time videoconferencing, telephone, email)	Primary measures: Staff outcomes – assessment of the programme's implementation, design and delivery from the preceptors’ perspective
	Level IV	Rural: Accessibility/Remoteness Index of Australia (ARIA) categories 1 to 6.	Contextual information: Correct use of technology and ability to use technology; willingness of participant to undertake self-directed learning	Positive impact: Interactive elements of the online programme, such as reflective exercises, were useful for learning
		Australia		Considerations: Some IT issues. Introductory video would be useful for programme but weekend course or videoconferencing is a better mode of delivery*.* Telephone helpline would be useful Limitations: Presumed pharmacists were good self-directed learners and had adequate IT skills
Gardner *et al*. [63]	Non-experimental – descriptive post-evaluation	Nurses	Intervention: Professional support, training and education; supporting nurses in rural areas to understand and conduct research (combination: face to face, videoconferencing, telephone, email)	Primary measure: Staff outcomes – orientation to research
	Level IV	Rural and remote	Contextual information: External support; accessible and adequate resources (all participants had access to necessary resources; textbooks and resource packages were provided as well as access to computers during the workshops); active involvement of participants (content of programme was responsive to the needs of the nurses at the rural and remote sites); networking and relationships (mentorship and collaboration encouraged)	No impact: the survey results do not demonstrate any major changes over time in perceived knowledge of research, research orientation or perceptions of barriers and supports to research. Despite the same structured educational intervention being delivered at two rural sites, clinical nurses at only one site completed the research proposals within the study timeframe
		Australia		
Hoon *et al*. [64]	Mixed methods – before and after design, action research	Nurses and medical practitioners	Intervention: Training, education and mentoring; information on how a training programme was developed: planned and delivered in collaboration with local community partners (face to face)	Primary measures: Staff outcomes – knowledge and skills in the delivery of chemotherapy and cancer care education. Service outcomes – connection between local rural health services and one or more of the urban specialist cancer services
	Level IV	Rural	Contextual information: Needs analysis; time relief to attend five-day placement; funding to attend five-day placement (salary costs of rural participants, travel and accommodation, salary funding for mentor for one day of placement); indemnity, legal matters, duty of care, responsibility (hands-on opportunities limited by indemnity issues and issues from metro staff around relinquishing cancer care to practitioners with little time, knowledge or skill)	Positive impact: Post-programme significant improvement in understanding of principles of chemo delivery including some technical details; improved confidence in technical details; knowledge translation to other rural practitioners and organisations; changes in procedures and practices; isolated incidences of improved client care (less travel for clients) Considerations: Programme was limited by unmet expectations; integrating new practices with already demanding practice; quality and safety issues as perceived by metro teachers and mentors; variability in opportunities (for example some hands-on but some not, some mentoring but some not)
		Australia		
MacKinnon [65]	Qualitative – institutional ethnography	Nurses	Intervention: Professional support, training and education; exploration of nurses’ experiences of learning to provide maternity care in rural settings (mode not specified)	Primary measures: Staff outcomes – behaviour, practice, knowledge, skills, job satisfaction
				Secondary measures: Patient outcomes – safe practice; service outcomes – quality
	Level I	Rural: less than 10,000 people living beyond commuting distance of an urban setting	Contextual information: External support (example of funding provided to one participant to upskill in maternity care in a regional centre); accessible and adequate resources; networking and relationships	Difficult to learn about maternity in small rural hospitals, in an environment where few staff members are available and little education is provided; concerns expressed about remaining ‘experienced’ and retaining newly acquired skills; experienced nurses had been mentored to ‘learn maternity’ by an experienced maternity nurse; however, birth rates and staffing levels have changed and such practices as mentorship were no longer available for new RNs; going to a big city to learn maternity nursing ‘does not work’ because a rural hospital nurse is not able to access all the ‘fancy teams’ and high-tech equipment available to RNs working in the city; family commitments made it difficult for them to leave their community for CPE
Mitchell *et al*. [66]	Mixed methods – post-intervention, action research	Mental health practitioners	Intervention: Professional support, training, education and supervision; telemedicine network established to deliver and receive educational material via videoconferencing facilities (non-face-to-face: multi-site real-time videoconferencing)	Primary measures: Staff outcomes – accessing the network, participation in the network, useful sessions, benefits (networking, peer support)
	Level III (Daly)	Rural and remote	Contextual information: Access to technology (type of technology – videoconferencing units; ensuring availability of units, ensuring adequate IT support, ensuring organisational support); organisational commitment and support; ensuing funding; ensuring time available for setup; timing of programme: ensuring flexibility of delivery for staff	Positive outcomes: ability to access second opinions; ability to access specialists; ability to book teleconsultations; ability to access supervision from Adelaide; improved networking and peer support; improved efficiency and travel costs; improved health service efficiency (due to enhanced knowledge), retention Considerations: Impediments included competition with other services for use of equipment; equipment breakdown; time required to set up a session; staff on rotating rosters not being available at a set time; difficulties with local organisational processes, including approvals; imperfect synchronisation of lip movement and audio in videoconferencing sessions; high cost of sessions involving multi-site videoconferencing
		Australia		
Owen *et al*. [67]	Non-experimental – pre- and post-intervention descriptive evaluation	Mental health practitioners	Intervention: Professional support, training and education; intermittent outreach service provided by metro mental health specialist practitioners to rural and remote areas – includes joint patient care sessions, education sessions and peer support (face to face)	Primary measures: Staff outcomes – clinical skills gained; success of education sessions; knowledge gained; attitudes. Service and client outcomes – admission rates from each town to a regional centre and transfer of clients for care to regional centres; prescription rates of psychotropic drugs from 18 months prior and during the project via Pharmaceutical Benefits Scheme data
	Level IV	Rural and remote	Contextual information: Active involvement of stakeholders in programme design and implementation (a representative steering committee to finalise teaching topics and oversee project comprising rural health staff, metropolitan health and education staff, rural health administration; clinics organised by local contact); external organisation of the project and intervention (research officers from the university coordinated and organised the project; project lead was a visiting specialist with a vested interest in the programme being successful); marketing of programme (flyers sent to promote education sessions; project promoted in multiple mental health venues; CVs of visiting team circulated); funding (transport costs were met by the project but salaried visiting staff were ‘donated’ to the project)	Positive impact: Education session evaluation – perceived increase in knowledge by most participants; content was perceived as relevant, appropriate. Regional admission rate increased and prescriptions increased (admission rates and prescription rates not controlled statistically for any other factors so cannot attribute to the intervention per se*).*
		Far west New South Wales, Australia		Less impact: Knowledge assessment – correct responses to mental health statements same prior to and after intervention (no change from baseline – but possibly using a poor measurement tool); before and after skills assessment (clinical vignettes); small improvement in ability to diagnose psychiatric conditions
Schopp *et al*. [68]	Non-experimental – descriptive pre-and post-test evaluation	Psychologists	Intervention: Professional support, training and education; specialist one-on-one support and training for remote generalist psychology clinicians through telehealth videoconferencing and website support for families (non-face-to-face: real-time videoconferencing)	Primary measures: Staff outcomes – knowledge gains: rural clinicians undertook a pre-test on issues related to TBI that was matched to the training content Patient outcomes – client satisfaction, family access via structured interview
	Level IV	Rural: Mid-Western rural communities	Contextual information: Access to technology (with ability to encrypt and decrypt data for patient confidentiality); externally supported and organised (participating rural practitioners, technology, content of sessions); attributes of teacher (approachable)	Positive impact: Significant pre- and post-test scores for clinicians for knowledge gain (and self-reported confidence) (means not given). Patients found trained clinicians helpful and knowledgeable. Compared to the 11 patients who chose not to use the trained clinician, authors report trained providers were perceived as more helpful and more knowledgeable than untrained providers – this was reported as significant (the statistical analyses of patient responses when comparing trained with untrained clinicians is flawed, thus we cannot rely on these results)
		USA		
Sullivan *et al*. [69]	Non-experimental – descriptive post-intervention evaluation	Medical practitioners (GPs and psychiatrists)	Intervention: Training, education and mentoring; shared care strategies between expert mentor and GP via telephone combined with monthly education sessions and joint clinical consultation (combination: face to face and telephone)	Primary measures: Staff outcomes – identify key success factors to shared care in this manner – measured one year after the pilot project
	Level IV	Rural	Contextual information: Attributes of teacher (relaxed, expert did not take on teacher role, mentor, approachable); needs analysis; accessible resources (funding for travel to education sessions, time to attend sessions)	Positive impact: Mentoring: All physicians viewed mentoring as highly valuable and a preferred method for accessing advice; allowed them to continue their own clinical interventions confidently, which they would not be able to support otherwise. Education: More satisfied if content relevant and if teacher utilised a relaxed approach to teaching
		Canada		
Tumosa *et al*. [70]	Non-experimental – descriptive pre- and post-evaluation	Multi-disciplinary	Intervention: Mentoring, training and education; evaluation of a geriatric scholar programme for rural primary care providers consisting of education and training in geriatrics and gerontology and in quality improvement (combination: face to face (clinical practice), webinars, audio conferences)	Primary measures: Staff outcomes – practice behaviour, knowledge, skills, ‘usefulness of programme’ Secondary measures: Service outcomes – quality improvement, perceived impact on patient care
	Level IV	Community of 5,000, 60 miles from major metropolitan areas.	Contextual information: Active involvement of stakeholders; organisational commitment; needs analysis (educational needs assessment); external support (financially and organisationally supported by networks of services with a ‘hub site’ located in a metro centre); accessible and adequate resources (intranet web-based platform to share resources as a learning community); networking and relationships; ongoing evaluation and feedback opportunities (identification of additional learning resources)	Positive impact: Improvements in self-reported competence and self-confidence in geriatric skills, topics and knowledge (and a resulting perceived change in practice); decline in continuing need for further education; high completion rates of QI projects; development of a rich learning community
		Rural Kentucky, USA		
**Papers specifically examining mode of delivery**				
Gagnon and Minguet [71]	Non-experimental – pre- and post-test pilot study evaluation	Medical practitioners	Intervention: Professional support, training and education; use of internet for delivery of online courses and collaboration with online tutorial sessions delivered twice weekly (non-face-to-face: virtual classes, collaborative web conferencing, real-time chat)	Primary measures: Staff outcomes – gain in knowledge
				Secondary measures: Staff outcomes – level of comfort with IT
	Level IV	Rural and remote	Contextual information: Access to technology (internet, computer, interactive IT programmes, webcam, microphone, software development, running and analysis); attributes of coach (availability); structure and content of programme (two tutorial sessions per week with real-time conversations online, virtual classes with real-time chatting and asynchronous exchange); correct use of technology and ability to use technology; external support and organisation	Positive impact: Reported the experience had brought them out of isolation and enabled very productive contacts with peers; participants likely to gain pedagogic knowledge and to maintain this knowledge over time
		Canada (Quebec); France		Less impact: Perception of level of comfort with information and communication technologies was unlikely to change
Stewart and Carpenter [72]	Qualitative – action research	Physiotherapists	Intervention: Twice weekly iChat with mentor and monthly videoconferencing with mentor and other mentees for three months (non-face-to-face: asynchronous chat, iChat, email, real-time videoconferencing)	Primary measures: Staff outcomes – effectiveness of mentoring using this medium; experience with technology
	Level III	Rural	Contextual information: Active involvement of stakeholders in programme design and implementation (measuring success and evaluating effectiveness of programme at key intervals and changing programme in response to feedback ); access to technology (Mac laptops with appropriate programmes; IT support; easy to use); mentor and mentee attributes (relationship between mentor and mentee); externally organised and supported	Positive impact: Improved communication (iChat sessions replicated the colleague interaction that was generally missed in sole positions); improved clinical reasoning, confidence and knowledge translation
		Canada		
Chipps *et al*. [73]	Non-experimental – descriptive pre- and post-evaluation	Medical practitioners (psychiatry)	Intervention: Videoconference-based psychiatry registrar training programme (non-face-to-face: real-time videoconferencing)	Primary measures: Attendance; familiarity with videoconferencing; cost and time savings; appropriateness of content and mode, technical issues
	Level IV	Urban and rural	Contextual information: Accessible and adequate resources; networking and relationships (videoconferencing was real time with participant interaction)	Positive outcomes: Improved access to education (increased attendance and reduction in travel resulting in time and cost savings); videoconferencing perceived as appropriate educational tool (and as effective as face-to-face teaching); videoconferencing gave satisfactory interaction
		South Africa		Considerations: technical issues audio quality
Brownlee *et al*. [15]	Qualitative – thematic analysis of interviews	Social workers	Intervention: Perception of utility of different technologies for supporting social work practice in rural areas (non-face-to-face: chat, email, internet, videoconferencing)	Primary measures: Staff outcomes – use of technology, change in practice behaviour, connectedness
	Level III	Rural and remote: Practitioners from areas where the population densities are well below 400 people/km^2^	Contextual information: Accessible and adequate resources (internet access, email, caseload database systems, phone systems, for example, telehealth)	Positive impact: Professional networking; clinical feedback; supervision and access to services seem to have increased with the availability and use of the internet
		Canada		Considerations: Not all use internet – language barriers; cumbersome and confusing; not all challenges of rural practice have been remedied, or even affected, by the internet – for example dual relationships in small rural towns

**Table 4:** Nature of the literature

**Component**	**Number of papers**
Intervention	
Training and education^a^	20
Combination	9
Professional support	4
Supervision	4
Technology focus	4
Mentoring	2
Professional groups	
Nursing	9
Allied health	5
Pharmacists	(1)
Physiotherapists	(2)
Psychologists	(1)
Social workers	(1)
Medics and general practitioners	8
Multi-disciplinary (>3 professions)	8
Other^b^	5
Combination	5
Nursing + dietitians	(1)
Nursing + general practitioner/medic	(2)
Mental health practitioner + general practitioner	(1)
Psychologists + social workers	(1)
Mental health practitioners	3
Country	
Australia	20
Canada	10
United States of America	7
Guatemala	1
Kenya	1
New Zealand	1
South Africa	1
United Kingdom	1

^a^Training and education: Continuing Professional Education (CPE), Continuing Medical Education (CME), Continuing Professional Development (CPD); ^b^administrative staff, palliative carers, Aboriginal health workers, health workers, community members, spouses.

### Evidence strength

The strength of evidence was, overall, low with the majority of studies being either Level IV (NHMRC, quantitative evidence) or Level III (Daly *et al*., qualitative evidence) (Table 5). One randomised controlled trial (Level II) [42] and two Level I qualitative studies were identified [35,65].

**Table 5:** Summary of outcomes utilised to characterise success

**Outcome**	**Studies examining outcome**
*Service outcomes*	
Perceived improvement in quality, safety of care or higher standards of practice	15, 39, 41, 47, 49, 54, 70, 71, 77
Change in organisational culture	34, 36, 37, 44
Improvement in access to care and clinic efficiency (perceived)	41 53, 56, 76
Improved ‘actioning’ of issues	33, 34
Increased certification of practitioners	74
Cost and time savings	71
Reduction in travel	71
Improved referral pathways, connections or collaboration	66
*Staff (including programme) outcomes*	
Increases in self-reported knowledge, confidence and competence in practice, skills and/or clinical decision-making	15, 33, 41, 44, 46, 48, 49, 52, 54, 59, 60, 61, 71, 72, 75, 76
Content or structure of material or programme perceived as appropriate and relevant by participants	44-46, 49, 53, 54
Retention of staff	39, 40, 49, 66, 75
Greater understanding of role of mentoring or supervision	37, 36, 44
Capacity to attend or participate in programme	49, 52
Improved collaboration with other health workers	41
Knowledge gains	14, 34
Improved attitude towards supervision or mentoring	36, 37
Job satisfaction	15, 71
Improved staff well-being	43
Improved knowledge of roles and knowledge sharing	54
Improved reflective practice	66
*Programme outcomes*	
Attendance and completion rates	14, 33, 34, 36, 37, 43, 45, 54, 57, 59, 72, 76
Sustainability of programme	34, 36, 37, 43, 47, 48, 54, 56
Knowledge translation to other rural practitioners and organisations	48, 54, 76
Engagement with the programme	33, 54, 59
Satisfaction with the programme (including perceived positive use of staff time)	33, 52, 66
Attainment of learning goals	49, 52
*Patient outcomes*	
Staff perceived improvements in screening procedures	41
Reduced admission rates from a rural to a regional centre and reduced transfer of clients for care to regional centres	53, 76
Client satisfaction with services	61
Improved quality and completeness of health information and services received by clients	56

The research was predominantly descriptive pre- and post-intervention evaluations using unvalidated questionnaires (Table 3). The most informative qualitative studies explored mechanisms for successful support interventions. For example, Lynch and Happell [33,34] examined the ‘process and journey’ of a clinical supervision implementation strategy for mental health workers in a rural health service.

### Support interventions

Interventions were predominantly training and education programmes delivered face to face, remotely or utilising a combination of face-to-face delivery with technology. There were a limited number of evaluations specifically exploring supervision and mentoring interventions (*n* = 5). A number of papers explored a combination of support strategies (Tables 3 and 4).

Four categories of support were identified from the literature (Table 3), generally reflecting the key search terms. These included supervision strategies or interventions, professional support strategies, training and education interventions and mentoring strategies or interventions.

*Supervision* incorporates interventions primarily focussing on the delivery of a supervision programme or the implementation of a supervision strategy.

*Training and education* includes interventions involving training in a particular skill, for example, online training programmes for rural and remote mental health practitioners in cognitive behavioural therapy (CBT) [42], CPE, CME or CPD.

*Professional support* includes interventions that aim to support practitioners through ‘connectedness’ using networking and collaboration opportunities. It also includes strategies such as supporting practitioners to participate in planning to improve working conditions (for example, see Teasley *et al*. [38]) and changes in work structures to support practitioners to undertake support opportunities (for example, see Healey-Ogden [40]).

*Mentoring* includes interventions where the delivery of a mentoring programme was the primary focus of the paper.

There was also a collection of studies that specifically examined the mode of delivery of an intervention rather than the intervention *per se*. These studies are outlined in Table 3 and are explored further (below) as a contextual mechanism.

### Defining success: measures used to examine the effect of support interventions on patient, staff and service outcomes

Successful support interventions were found to be those that positively influence or enhance patient, service and/or staff outcomes. Table 3 describes the outcomes measured for each study and Table 5 summarises the outcomes used to characterise success.

Staff outcomes were measured most frequently, generally through self-report measures such as self-reported gains in knowledge, competence and skill and/or clinical practice. Other staff outcomes included: gains in knowledge (tested via knowledge tests), feelings of isolation or socialisation (for example, connectedness with other centres or colleagues), levels of information exchange and networking, and retention (intention to leave and staff turnover).

Programme outcomes were also examined and included satisfaction with the programme (relevance, ease of use, ease of access and fulfilling needs), attendance and participation levels and numbers, and level of comfort or competence with technology.

Service outcomes were most frequently reported around perceived improvement in quality, safety of care and higher standards of practice. These outcomes were often also cited as patient outcomes. Other service outcomes included changes in the organisational culture and improvements in access to care and clinic efficiency.

There were four cases where patient outcomes were reported. These included staff perceptions of improvements in screening procedures for clients, improved access to and quality of services, reduced number of transfers of care from rural to regional centres, improved client satisfaction with services and the completeness of health information and services received by clients.

### Mechanisms specific to rural and remote contexts and their relation to outcomes

**Figure 2:**
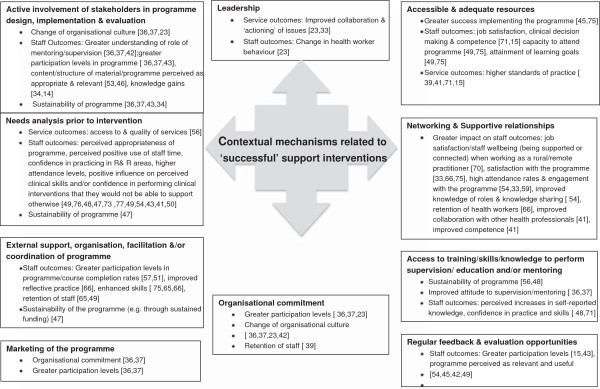
Mind map of key mechanisms and their relation to outcomes.

A total of ten mechanisms were identified. These are outlined in Table 6. The mind map of the relation between mechanisms and outcomes is illustrated in Figure 2.

**Table 6:** Key mechanisms identified from synthesis of evidence

**Evidence (reference)**	**Active involvement of stakeholders**	**Organisational commitment**	**Access to training, skills or knowledge for the intervention**	**Needs analysis**	**External support**	**Regular feedback and evaluation opportunities**	**Marketing of the programme**	**Accessible and adequate resources**	**Networking and relations**
Brambila *et al*. [44]	√			√	√			√	
Buckley *et al*. [45]								√	
Butcher [60]			√	√	√			√	
Conger and Plager [37]								√	√
Cunningham *et al*. [47]		√							
Dalton *et al*. [62]			√						
D'Souza [48]								√	
Gagnon and Minguet [71]								√	
Gibb *et al*. [61]	√			√	√	√			
Glazebrook *et al*. [50]					√				√
Haythornthwaite [52]					√			√	
Hoon *et al*. [64]				√					
Kelley *et al*. [54]			√						√
King *et al*. [53]			√			√			
Lynch and Happell [33,34]	√	√	√	√		√	√		
Mitchell *et al*. [66]		√							
Newman *et al*. [56]				√					
Owen *et al*. [67]	√				√		√		
Schoo *et al*. [57]	√			√	√	√			
Schopp *et al*. [68]					√			√	
Stewart and Carpenter [72]	√					√		√	√
Sullivan *et al*. [69]				√					
Teasley *et al*. [38]	√								
White *et al*. [58]				√				√	
Xavier *et al*. [36]				√	√			√	
Arora *et al*. [41]				√	√	√		√	√
Bennett-Levy *et al*. [42]					√			√	√
Blattner *et al*. [43]			√					√	
Brownlee *et al*. [15]								√	
Cameron *et al*. [39]	√								√
Chipps *et al*. [73]								√	√
Church *et al*. [46]					√				√
Doorenbos *et al*. [14]	√			√	√		√	√	
Ellis and Philip [49]					√			√	
English *et al*. [35]	√	√		√	√				√
Gardner *et al*. [75]					√			√	√
Gorsche and Woloschuk [51]					√			√	
Healey-Ogden *et al*. [40]		√		√	√			√	
Koczwara *et al*. [55]			√	√		√	√	√	√
MacKinnon [65]					√			√	√
Tumosa *et al*. [70]	√	√		√	√			√	√
Wright *et al*. [59]				√		√		√	√

#### ***Conducting a needs analysis prior to intervention***

White *et al*. [58] described a government-run CME programme where an annual educational needs analysis questionnaire was distributed to all rural and remote general practitioners (GPs) to inform them of the programme. The authors reported that access to CME contributes to confidence in practising in rural and remote areas, CME strongly alleviates professional isolation and GPs are less likely to remain in practice without access to CME.

On a more individual level, Tumosa *et al*. [70] described a programme of rural education that requires participants to complete a survey to assess individual educational needs. The needs analysis was used to then design an appropriate mix of clinical, didactic, supervised and administrative learning experiences. Participants reported high levels of educational goal achievement from participation in the programme.

A needs analysis prior to intervention was linked to the following outcomes: improved service outcomes including improved access to services, improved quality of services and more sustainable programmes; and positive staff outcomes such as perceived appropriateness of programme, perceived positive use of staff time, confidence in practicing in rural and remote areas, higher programme attendance levels, positive influence on perceived clinical skills and confidence in performing clinical interventions that they would not otherwise be able to support.

#### ***Active involvement of stakeholders in programme design, implementation and evaluation***

Gibb *et al*. [61] used an action research approach to develop an organisational definition of mentoring and to identify the qualities valued in a mentor. The findings from this action cycle were converted into questionnaires by a research team. The results of the questionnaires were used to develop a set of guidelines regarding qualities desired in mentors and mentees, as well as an evaluation tool for monitoring the mentoring relationship.

Actively involving stakeholders in programme design, course or programme content, implementation and evaluation was linked to: positive changes in organisational culture towards supervision, a better understanding of the role of mentoring and supervision, higher participation levels in the programme, a perception that the content and structure of the material or programme were appropriate and relevant, higher and sustained attendance and increased sustainability of the programme.

#### ***External support, organisation, facilitation and/or coordination of programme***

Action research, for example, involving support from university researchers can contribute to successful outcomes. One study describes how university researchers assisted a service to implement a continuing education (CE) programme for physiotherapists in rural Victoria, Australia [57]. Researchers developed a questionnaire to assess the CE needs of physiotherapists. The results then informed the development of a CE programme and programme evaluation tools. All targets for success (as identified and defined by stakeholders) were reached.

External support, organisation, facilitation and/or coordination of the programme were linked to the following outcomes: good programme attendance rates and successful knowledge translation between colleagues.

#### ***Organisational commitment and leadership***

Strong organisational commitment has been linked to: greater participation levels, change in organisational culture, sustainable programmes and improved patient outcomes and quality of service provision. Leadership and organisational commitment to a newly developed supervision programme, as demonstrated by a senior management team forming a clinical supervision committee to oversee implementation and evaluation of the staff-led supervision programme [33,34], was reported to lead to a large change in culture in relation to supervision within the mental health programme. The successful adoption of best practices in rural Kenyan hospitals was related to the ‘implementing team, hospital management, and facilitator together provided leadership and supported a shift in organisational culture and commitment that helped motivate health workers and change their individual behaviours’ (p. 4) [35].

#### ***Accessible and adequate resources***

Having access to adequate and appropriate resources (including time) to undertake or provide support programmes has been linked to greater success implementing support programmes and potentially retention of staff.

Significantly greater retention rates for rural medical practitioners have been achieved through the provision of fully subsidised locum-relieved training programmes [51]. Greater retention of nursing staff following the implementation of an 80/20 staffing model in a rural hospital has also been reported [40]. The 80/20 model provides staff with 20% of their salaried time off from direct patient care in order to pursue various types of professional development activities. Importantly backfill positions were created to accommodate the 20% reduction in clinical duties.

Having access to adequate and appropriate resources was also linked to high levels of staff satisfaction with the intervention or programme and the mode of delivery of the intervention.

#### ***Mode of delivery, format and timing***

Perceptions of greater programme success were associated with a mentoring programme when three key elements were addressed: timing (twice weekly ‘chat’ with monthly videoconference); mode of delivery (iChat, videoconference and email); and format (‘chat’ mentoring weekly and videoconferencing where mentees take turns) [72]. It was perceived, for example, that electronic iChat sessions replicated the colleague interaction that was generally missed in sole positions.

Where the format of a support programme included opportunity for interaction, networking and/or peer support, there was a relation with successful outcomes such as reduced feelings of isolation, high access rates, completion of and participant satisfaction with the programme. This was the case for both face-to-face [46,47,54,59] and non-face-to-face interventions [72].

A randomised controlled trial examining the impact of an accredited online training program in CBT for rural and metropolitan psychologists, compared structured online modules of study with or without support from a CBT expert [42]. The supported training group was significantly more likely to finish all training modules than the group that undertook the training without support. An online resource for rural health-care practitioners that was supplemented by online, facilitated modules also demonstrated high access rates [55].

Where the mode of delivery was face to face, provision of funding and support (for travel, accommodation, salary, time and locum relief [50-52,58,65,67]) related to successful outcomes including sustainability of (and ability to conduct) the programme and participation rates. This was also the case for non-face-to-face interventions where funding was essential to equipment provision and payment of participating specialists, teachers, mentors and supervisors [41,42,66].

For face-to-face interventions, where the programme was delivered, for example in a local or regional centre [44,50,54,57,65], was also important, relating to successful outcomes such as capacity to attend the programme.

When interventions were delivered remotely utilising technology, the following elements were identified that related to successful outcomes: flexibility in the timing of delivery [15,48,59,66]; adequate preparation for technology to work [56], such as an orientation to the technology and online learning approach [41]; external support and coordination (including organisation of technology and participants, and development or organisation of the content of sessions) [14,36,41,46,55,56,68,71-73]; ease of use of technology (including adequate connection speeds) [15,41,42,52,55,56,59,62],[66,71,73,74]; correct use of technology and ability to use technology [41,45,46,52,62,71,73]; confidential transmission of patient details, information and case histories [41,45,66,68,72]; and willingness of participants to undertake self-directed learning [42,59,62]. These elements related to success factors including the sustainability of the programme, participation rates and participant satisfaction levels.

The importance of addressing these elements was illustrated by Mitchell *et al*. [66], who reported satisfaction with and the overall success of technologically driven interventions can be impeded by: competition with other services for use of the equipment; equipment breakdown; the time required to set up a session; staff on rotating rosters not being available at a set time; difficulties with local organisational processes, including approvals; the imperfect synchronisation of lip movement and audio in videoconferencing sessions; and the high cost of sessions involving multi-site videoconferencing. These mechanisms are important for a successful online format.

#### ***Access to training, skills or knowledge for supervision, education or mentoring***

Ensuring access to training, skills or knowledge for supervision, education, training or mentoring has been linked to the sustainability of a programme, an improved attitude to supervision or mentoring and an increased effect on staff outcomes (perceived increases in self-reported knowledge, confidence in practice and skills). One element of a successful staff-led supervision programme was to ensure that all supervisors and supervisees received external training in supervision [33,34].

#### ***Regular feedback and evaluation of the programme***

Regular feedback and evaluation of support programmes has been linked to improved knowledge translation, sustainability of the programme and greater effect on staff outcomes. The importance of measuring success and evaluating the effectiveness of a programme at key intervals and changing the programme in response to feedback was demonstrated in a study of electronic mentoring of rural paediatric physiotherapists [72]. The study demonstrated improved communication between mentor and mentees and improved clinical reasoning, confidence and knowledge translation.

#### ***Marketing of the programme***

Officially launching a supervision programme, as described in two studies, had a twofold effect: (i) it demonstrated organisational commitment and (ii) it increased awareness of and participation in the programme [33,34].

#### ***Networking and supportive relationships***

Networking and supportive relationships refer to networking opportunities, peer relationships, relationships with experts and specialists and relationships with the community. They are linked with high levels of participant satisfaction with the intervention or programme, greater attendance rates, improved knowledge of roles, retention of health workers, improved quality or safety of practice and improved reflective practice.

Retention of GPs for longer than a four-year period across four rural communities in Canada was found to be related to community factors such as appreciation shown by the community for the practitioner and community connection or a sense of belonging and integration into the community [39]. The absence of supportive relationships has been related to declining birth rates in rural areas, because there were fewer trained staff to provide maternity services. One study describes how scant access to birthing experiences and therefore experienced mentors for new nurses to gain this experience, restricts access to maternity services for rural clients [65].

## Discussion

This synthesis has identified a number of support interventions for health-care practitioners in rural and remote contexts, the outcomes that such interventions can generate and has identified mechanisms, specific to rural and remote contexts, that relate to successful outcomes for staff, patients and services.

We identified that the outcomes of support interventions for practitioners in rural and remote contexts may be enhanced if the support strategy includes: consultation with staff prior to the programme to assess individual, collective and context specific needs; external support; accessible and adequate resources assisting staff to undertake or access the programme; and interactive and networking opportunities.

Professional networking, education and supervision opportunities for rural and remote health-care practitioners have increased with the availability and use of the internet [15]. We found that for programmes delivered remotely using technology, outcomes such as engagement with the programme, reduction of feelings of isolation, achievement of learning outcomes and knowledge gains and participant satisfaction may be further enhanced if there is a ‘human element’ to the programme, such as networking opportunities, online facilitation and/or interactive learning elements. Interactive techniques have been shown elsewhere to be the most effective educational technique for changing physician care and influencing patient outcomes [8]. However, to gain such benefits, the format and timing of the technologically driven support strategy also need to be carefully considered such that they are user friendly and flexible enough to be accessed by participants at convenient times.

Most importantly, in rural and remote contexts the evidence suggests that supporting practitioners to access support interventions by means of financial reimbursement, travel subsidies, backfilling and organisational commitment can directly or indirectly influence retention of staff and the quality and safety of services.

A recent meta-synthesis of recruitment and retention of occupational therapists and physiotherapists in rural regions supports these findings. Support from the organisation influences retention and with support, challenges can become rewards and assets [17]. These findings are also consistent with Humphreys and colleagues’ research examining the relation between education, training and retention of the rural primary health-care workforce [23]. Furthermore it has been demonstrated that without organisational commitment, efforts to change clinical practice by influencing individuals is ineffective [75].

This review has attempted to capture the complexity of the mechanisms required in a rural and remote context to operationalise a successful support intervention for health-care practitioners. We therefore chose not to exclude research on the basis of quality, opting instead to extract a fuller understanding of ‘relationships, mechanisms and meaning’ within the evidence base [27]. This form of exploration is something a traditional systematic review is limited in performing [31], particularly in rural and remote contexts [20], despite or because of inclusion of high-quality research. As such, both approaches have their limitations.

The most rigorous sources of evidence included in this review were also the leanest on contextual and mechanism data. For example, Gorsche and Woloschuk conducted a longitudinal matched case–control study [51] that importantly found that retention of rural and remote GPs can be significantly enhanced through provision of training. The mechanisms that produced this result, however, are not clear. On the other hand, Healey-Ogden *et al.*[40] described a number of mechanisms that lend support to the premise that financially supporting professional development opportunities can lead to retention of staff; however, the study is of low quality.

There is an inherent difficultly therefore in balancing scientific rigour with identification, exploration and reporting of contextual elements that may influence the outcome of a support intervention in a complex context such as the rural and remote health-care environment.

### Study limitations

There was a dominance of literature pertaining to education and training interventions and a dearth of literature evaluating support, supervision and mentoring interventions. The mechanisms identified in this review may, therefore, not reflect the entirety of mechanisms required for successfully supporting health practitioners in rural and remote health-care contexts.

This limitation may have been partially addressed through the undertaking of additional hand searches of cited reference lists or searches within the grey literature. Neither of these strategies, however, were undertaken for this review.

Measures of success in this review have been influenced by the nature of the research methodologies and corresponding measurement tools employed by the reviewed papers. There was an overrepresentation, for example, of papers that measured the success of an intervention in terms of self-reported staff outcomes such as knowledge, skill or confidence gains utilising unvalidated questionnaires.

The review has focussed on identifying relations between contexts, mechanisms and outcomes. Although an integrative review methodology and thematic analysis were employed, further research investigating these relations may be strengthened by the use of inductive logic reasoning [76]. This combines programme logic [30], realistic evaluation [27] and other structure-process-outcome models to extract and organise the data systematically under the headings: drivers, contexts, mechanisms (barriers and facilitators), outputs and outcomes.

### Further research

Despite the importance of enabling and facilitating access to support for health-care practitioners in rural and remote contexts, the capacity of a practitioner to access a support intervention was rarely used as a measure of success nor were the factors that facilitated or hindered a practitioner from accessing support explored. Attendance rates or completion rates of the intervention were proxies. There was also little information on the effect of supervision interventions on any outcomes. Equally, only four papers identified the retention of health workers as an outcome of interest.

## Conclusion

Through synthesis of the literature, this research has identified a number of key mechanisms that are associated with successful support interventions for health-care practitioners in rural and remote health-care contexts. In particular, there is a need for health-care organisations to make a commitment to actively enable practitioners in rural and remote contexts to access support interventions.

This review has identified a need for better quality research, in particular research assessing supervision interventions and retention as an outcome of support strategies, to enable more concrete conclusions to be drawn regarding the direct effect of support interventions for rural and remote health-care practitioners on staff, patient and service outcomes.

## Abbreviations

ARIA: Accessibility/Remoteness Index of Australia; CBT: cognitive behavioural therapy; CE: continuing education; CME: continuing medical education; CPD: continuing professional development; CPE: continuing professional education; GP: general practitioner; NHMRC: National Health and Medical Research Council (of Australia); RRMA: Rural Remote and Metropolitan Areas; WHO: World Health Organisation.

## Competing interests

The authors declare that they have no competing interests.

## Authors’ contributions

JY carried out the literature searches. AM and JY carried out the initial screening process. AM, JC, RP, DB and JY screened all papers for inclusion. AM carried out the thematic analysis and wrote the paper. SN assisted with thematic analysis and advised on the theoretical approach. AM conceived the study and drafted the initial manuscript. JC, RP, DB, JY and SN participated in its design and coordination and helped to draft and review the manuscript. All authors read and approved the final manuscript.
